# The relationship between physical activity self-worth and body appreciation among physically active women: the mediating role of psychological resilience

**DOI:** 10.3389/fpsyg.2026.1790071

**Published:** 2026-03-02

**Authors:** Serkan Kabak, Ozkan Isik, Laurentiu-Gabriel Talaghir, Dumitru Marius Cosoreanu

**Affiliations:** 1 Faculty of Sport Sciences, Trabzon University, Trabzon, Türkiye; 2 Faculty of Sport Sciences, Balikesir University, Balikesir, Türkiye; 3 Directorate of Sports Sciences Application and Research Center, Balikesir University, Balikesir, Türkiye; 4 Faculty of Physical Education and Sport, Dunarea de Jos University of Galati, Galati, Romania

**Keywords:** body appreciation, mediation role, physical activity self-worth, psychological resilience, women

## Abstract

**Background:**

Physical activity self-worth reflects the value women attribute to themselves through being physically active and has been associated with more positive body-related outcomes. Psychological resilience may help explain this link by supporting adaptive coping and more constructive self-perceptions. In this study, the mediating role of psychological resilience in the relationship between physical activity self-worth and body appreciation was examined among physically active women.

**Method:**

A quantitative correlational design was used with 640 physically active women. In the study, women's physical activity self-worth, body appreciation, and psychological resilience were assessed using the Women's Physical Activity Self-Worth Scale, the Body Appreciation Scale, and the Brief Resilience Scale. Mediation was tested with Hayes' PROCESS macro (Model 4) using 5,000 bootstrap resamples, and direct, total, and indirect effects were evaluated via bootstrap confidence intervals.

**Results:**

Physical activity self-worth significantly predicted psychological resilience (*b* = 0.333; SE = 0.079; *p* < 0.001), and psychological resilience significantly predicted body appreciation (*b* = 0.309; SE = 0.037; *p* < 0.001). Physical activity self-worth had a significant total effect on body appreciation (*b* = 0.688; SE = 0.088; *p* < 0.001) and remained significant after including psychological resilience (direct effect: *b* = 0.585; SE = 0.088; *p* < 0.001). Additionally, Bootstrap analyses indicated a significant indirect effect via psychological resilience [a^*^b = 0.103; 95% CI (0.053, 0.158)].

**Conclusion:**

Psychological resilience mediates the association between physical activity self-worth and body appreciation in physically active women, indicating that resilience-related processes are relevant for understanding and supporting positive body-related evaluations.

## 1 Introduction

Today, women are constantly confronted with the ideal body image presented by the media, and this negatively affects their process of evaluating and accepting their own bodies (Çakir, 2020; Poorani, 2012). Sedentary lifestyles, aesthetic pressures, and societal expectations negatively impact women's satisfaction with their bodies and their self-worth regarding physical activity (Cuprika et al., 2018; Mihalko et al., 2001; Yadav, 2025). However, physical activity is a powerful tool for maintaining and even improving physical fitness (Greenberg et al., 2004; Faulkner and Biddle, 2001; Yeh et al., 2016). Regular participation in physical activities helps individuals feel competent, strong, and in control, reducing the emotional burden created by negative body image (Vurgun, 2015). However, the occurrence of these gains largely depends on the development of psychological resilience, and accordingly, increased self-worth toward physical activity can transform women's body appreciation into a more positive perception through psychological resilience.

Physical activity is becoming increasingly important for women in terms of self-worth and psychological factors related to body appreciation (Casuso-Holgado et al., 2024; Ruiz-Turrero et al., 2022; Walters et al., 2023). Previous studies have shown that positive self-worth related to physical activity supports women in feeling more competent, evaluating their bodies more positively, and improving their psychological wellbeing (Kaya et al., 2021; Vega et al., 2022; Vurgun, 2015). In this context, addressing the relationship between physical activity self-worth and body appreciation through psychological resilience can offer an approach that supports women's emotional resilience and individual wellbeing. Understanding the mechanisms by which psychological resilience influences the relationship between physical activity self-worth and body appreciation is critical for developing strategies to increase women's psychosocial resilience and improve their quality of life (Oh and Gill, 2018; Liao et al., 2023). Self-determination theory (Deci and Ryan, 1985) provides a suitable framework for explaining this relationship. According to the theory, satisfaction of the basic psychological needs for autonomy, competence, and relatedness supports the internalization and maintenance of health behaviors and is linked to more favorable psychological outcomes (Deci and Ryan, 2000; Ryan and Deci, 2017). Increased self-worth due to physical activity strengthens the competence dimension. In physical activity contexts, competence-related experiences (e.g., mastery, effectiveness, perceived control) are theorized to facilitate sustained engagement and adaptive psychological functioning (Hagger and Chatzisarantis, 2007; Ryan and Deci, 2017). As women can sustain physical activity, they may feel more successful, effective, and in control. Increased self-worth reduces negative evaluations of body image and supports the development of a positive self-image. Psychological resilience may function as a protective mechanism in this pathway by supporting adaptive coping and preserving self-evaluations under stress (Garmezy, 1991; Kobasa, 1979; Terzi, 2008). In turn, need-supportive motivational processes in physical activity settings have been linked to more positive body-related outcomes, suggesting a theoretically coherent basis for expecting physical activity self-worth—via resilience-related processes—to correspond with greater body appreciation (Hagger and Chatzisarantis, 2007; Sabiston et al., 2019).

In the 21st century, there has been a significant increase in the number of studies examining physical activity, body appreciation, and psychological resilience in women (Guszkowska, 2015; Izydorczyk et al., 2018; Kiliç and Yildirim, 2020; Seyrek et al., 2023; Siyahtas et al., 2025; Yalman and Gürsan, 2023; Yikilmaz et al., 2025). Especially, the literature contains findings suggesting that physical activity self-worth contributes to women feeling adequate, strong, and in control, thus reducing negative evaluations of their body image (Breault-Hood et al., 2025; Mosanya and Kassie, 2024; Streetman et al., 2023). Cao et al. (2024) reported that psychological resilience plays a protective role in the relationship between physical activity self-worth and body appreciation, and contributes to women's ability to cope more effectively with stress, anxiety, and external pressures. However, we found no studies that simultaneously examine physical activity self-worth, psychological resilience, and body appreciation within an integrative (holistic) mediation framework, in which physical activity self-worth is specified as the predictor, psychological resilience as the mediator, and body appreciation as the outcome. Therefore, evaluating the association between physical activity self-worth and body appreciation from a psychological resilience perspective may be critically important in addressing this gap.

This study aimed to reveal the mediating effect of psychological resilience on the relationship between physical activity self-worth and body image in women. In this context, the hypothesis of the study was that psychological resilience plays a significant mediating role in the relationship between physical activity self-worth and body appreciation in women.

## 2 Method

### 2.1 Research model

This study employed a quantitative research design. To derive generalizable conclusions from data collected from a sample representing a larger population, a general survey model was adopted (Karasar, 2012). Within this framework, the study was designed as a correlational survey to examine the relationships among physical activity self-worth, psychological resilience, and body appreciation in physically active women. Accordingly, the research model tested whether psychological resilience mediates the association between physical activity self-worth and body appreciation.

### 2.2 Study sample

The study sample comprised women attending pilates studios, zumba halls, and fitness centers in the Marmara Region of Türkiye (Istanbul, Kocaeli, Sakarya, and Balikesir). Data were collected using a convenience sampling approach between November 7 and December 23, 2025. Surveys were administered both in person (face-to-face) and via an online survey form. Eligibility for participation required (i) self-identifying as a woman, (ii) being 18 years of age or older, and (iii) currently attending a pilates studio, zumba hall, or fitness center in the Marmara Region during the data-collection period; additionally, participants were required to provide informed consent and to complete the questionnaire battery. A total of 750 women were approached and invited to participate; after applying the eligibility and data-quality criteria, 640 participants were retained for the final analyses. Prior to the final analyses, data integrity screening was conducted to ensure that only high-quality and analyzable cases were retained. Accordingly, cases were excluded if they exhibited substantial missing data on key study variables (i.e., incomplete responses that prevented computation of scale scores), or if response patterns indicated insufficient attention (e.g., uniform responding/straight-lining across multi-item scales or clearly repetitive patterned responses). In addition, when available, response submissions with implausible completion characteristics (e.g., extremely rapid completion suggestive of inattentive responding) were evaluated within the same data integrity framework and excluded if judged invalid. Participants' age ranged from 18 to 69 years (M = 27.69, SD = 9.51). According to Sekaran (1992), a sample size of 384 is considered adequate when the population size is up to 10,000,000; however, a larger sample (*N* = 640) was obtained to increase statistical power and strengthen the reliability of the findings. Regarding weekly physical activity participation, 19.7% of women reported being active 1 day per week (*n* = 126), 29.2% reported 2 days (*n* = 187), 22.5% reported 3 days (*n* = 144), 16.1% reported 4 days (*n* = 103), and 12.5% reported 5 days or more (*n* = 80) (Table 1).

**Table 1:** Demographic characteristics of the women.

**Variables**	***M***	**SD**
Age	27.69	9.51
**Weekly physical activity participation frequency**	***f***	**%**
1 day	126	19.7
2 days	187	29.2
3 days	144	22.5
4 days	103	16.1
5 days or more	80	12.5
		*N*: 640

### 2.3 Data collection tools

All participants were included in the study voluntarily, were informed in advance about the purpose of the study, and their consent was obtained following ethical principles. To collect data in the study, the Personal Information Form prepared by the researcher to determine the demographic characteristics of the participants, as well as the Women's Physical Activity Self-Worth Scale, Body Appreciation Scale, and the Brief Resilience Scale, were used. Each participant responded to the questions for an average of 10–15 min.

#### 2.3.1 Personal information form

The Personal Information Form will include descriptive items such as age and the number of days per week participants engage in physical activity.

#### 2.3.2 Women's physical activity self-worth scale

To assess women's physical activity self-worth, the Women's Physical Activity Self-Worth Scale developed by Huberty et al. (2013) and adapted into Turkish by Yurtçiçek and Kömürcü (2019) was used. The scale comprises 37 items rated on a 4-point Likert format (1 = Strongly disagree, 4 = Strongly agree) and includes three subdimensions: Knowledge Self-Worth (Items 1–16; 16 items), Emotional Self-Worth (Items 17–29; 13 items), and Social Self-Worth (Items 30–37; 8 items). The original study reported high internal consistency (Cronbach's alpha = 0.91) and acceptable split-half reliability (0.74), with subdimension alpha coefficients ranging from 0.80 to 0.89 (Yurtçiçek and Kömürcü, 2019). In the present study, internal consistency coefficients (Cronbach's alpha) ranged from 0.79 to 0.94.

#### 2.3.3 Body appreciation scale

To measure women's level of body appreciation, the Body Appreciation Scale developed by Tylka and Wood-Barcalow (2015) and adapted into Turkish by Anli et al. (2015) was used. The scale consists of 10 items rated on a 5-point Likert format (1 = Never, 5 = Always) and includes no reverse-coded items. A total body appreciation score is obtained by summing the item scores, with higher scores indicating greater body appreciation; possible scores range from 10 to 50. The Turkish adaptation reported high internal consistency (Cronbach's alpha = 0.94 for women and 0.93 for men) and strong test–retest reliability (0.90 for both genders), supporting the scale's reliability and validity (Anli et al., 2015). In the present study, the internal consistency coefficient (Cronbach's alpha) was 0.94.

#### 2.3.4 Brief resilience scale

The Brief Resilience Scale was developed by Smith et al. (2008), and its Turkish adaptation and psychometric evaluation were conducted by Dogan (2015). Item factor loadings range from 0.63 to 0.79, and item–total correlation coefficients range from 0.49 to 0.66. The scale includes 6 items rated on a 5-point Likert format: 1 = Not at all appropriate, 2 = Not appropriate, 3 = Somewhat appropriate, 4 = Appropriate, and 5 = Completely appropriate. Items 2, 4, and 6 are reverse-coded. Higher total scores indicate higher psychological resilience. In the Turkish adaptation study, the internal consistency coefficient (Cronbach's alpha) was reported as 0.83 (Dogan, 2015). In the present study, Cronbach's alpha was 0.76.

These Cronbach's alpha coefficients showed that the scales used in this study have adequate internal consistency (Salvucci et al., 1997).

### 2.4 Ethical approval

Ethical approval for this study was obtained from the Balikesir University Health Sciences Non-Interventional Research Ethics Committee (Decision Number: 2025/338). Participation was voluntary and written/electronic informed consent form was obtained from all women before data collection. Participants were informed of their right to withdraw at any time. Data were collected anonymously from women attending pilates studios, zumba halls, and fitness centers in the Marmara Region of Türkiye and were analyzed in aggregate to ensure confidentiality.

### 2.5 Statistical analysis

The data obtained were analyzed using IBM SPSS Statistics (v22). Before the main analyses, the dataset was examined for missing data and outliers, and descriptive statistics (mean, standard deviation, and minimum–maximum values) were computed for all variables. To assess the normality assumption, skewness and kurtosis coefficients were inspected and found to fall within the −2 to +2 range (George and Mallery, 2016). Accordingly, the data were considered suitable for parametric analyses. To test the mediating role of psychological resilience in the relationship between physical activity self-worth and body appreciation, a regression-based mediation analysis was conducted using Hayes' PROCESS macro (v4.2), Model 4 (simple mediation) (Hayes, 2013). The bootstrap method with 5,000 resamples was applied to estimate the indirect effect. The statistical significance of the mediation effect was determined based on whether the 95% confidence interval for the indirect effect included zero (Gürbüz, 2019; Hayes, 2013). Results were reported as unstandardized regression coefficients (b), standard errors, t-values, and *p-values* for each path, along with the explained variance (R^2^) for each model.

## 3 Results

Descriptive statistics presented in Table 2 indicate that the sample generally exhibits favorable psychological and body-related indicators. Participants' physical activity self-worth was *M* = 3.14 (SD = 0.37), which, given the scale range, suggests a relatively high level. Similarly, body appreciation was high (*M* = 4.14, SD = 0.74), whereas psychological resilience appeared to be at a moderate-to-high level (*M* = 3.30, SD = 0.75).

**Table 2:** Descriptive statistics for the main study variables.

**Variable**	***N***	**Min**	**Max**	**Mean**	**SD**	**Skewness**	**Kurtosis**
Physical activity self-worth	640	1.41	4.00	3.14	0.37	−0.300	0.863
Body appreciation	640	1.00	5.00	4.14	0.74	−0.820	0.429
Psychological resilience	640	1.00	5.00	3.30	0.75	0.001	0.490

**Figure 1:**
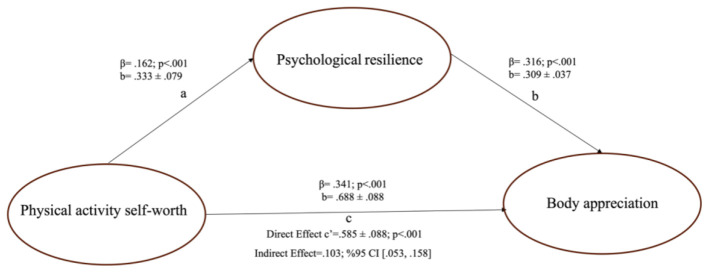
The mediating role of psychological resilience. **(a)** denotes the path from physical activity self-worth to psychological resilience; **(b)** denotes the path from psychological resilience to body appreciation (controlling for physical activity self-worth); **(c)** denotes the total effect of physical activity self-worth on body appreciation; **(c')** denotes the direct effect of physical activity self-worth on body appreciation after including psychological resilience.

The mediation model estimated with PROCESS Model 4 indicated that physical activity self-worth had a positive and statistically significant effect on psychological resilience (path a: *b* = 0.333; SE = 0.079; *t* = 4.206; *p* < 0.001). Psychological resilience, in turn, positively and significantly predicted body appreciation (path b: *b* = 0.309; SE = 0.037; *t* = 8.346; *p* < 0.001). The total effect model showed that physical activity self-worth significantly predicted body appreciation (path c: *b* = 0.688; SE = 0.088; *t* = 7.803; *p* < 0.001). When psychological resilience was included in the model, the direct effect of physical activity self-worth on body appreciation remained statistically significant (path c′: *b* = 0.585; SE = 0.088; *t* = 6.625; *p* < 0.001). Bootstrap resampling results further demonstrated that the indirect effect of physical activity self-worth on body appreciation through psychological resilience was statistically significant [a^*^b = 0.103; BootSE = 0.026; 95% CI (0.053, 0.158)], as the confidence interval did not include zero. Regarding explained variance, physical activity self-worth accounted for 2.6% of the variance in psychological resilience (*R*^2^ = 0.026), and physical activity self-worth together with psychological resilience explained 21.4% of the variance in body appreciation (*R*^2^ = 0.214) (Figure 1).

When the regression results of the mediation model presented in Table 3 are evaluated, physical activity self-worth significantly and positively predicts psychological resilience (path a: *b* = 0.333; *E* = 0.079; *p* < 0.001). In this model, physical activity self-worth explains approximately 3% of the variance in psychological resilience [*R*^2^ = 0.026; F_(1, 638)_ = 17.694; *p* < 0.001]. In the model where body appreciation was specified as the dependent variable, both physical activity self-worth positively and significantly predicted body appreciation (direct effect; path c': *b* = 0.585; SE = 0.088; *p* < 0.001) and psychological resilience had a positive and significant effect on body appreciation (path b: *b* = 0.309; SE = 0.037; *p* < 0.001). Together, these predictors explain approximately 21% of the variance in body appreciation [*R*^2^ = 0.214; F_(2, 637)_ = 74.237; *p* < 0.001]. Moreover, the total effect of physical activity self-worth on body appreciation was significant (path c: *b* = 0.688; SE = 0.088; *p* < 0.001). Bootstrap results (5,000 samples) showed that the indirect effect through psychological resilience was significant [a^*^b = 0.103; BootSE = 0.026; 95% CI (0.053, 0.158)], supporting the mediating role of psychological resilience.

**Table 3:** Regression analysis results for the mediation model.

	**Outcome variables**					
	**Psychological resilience (** ***M*** **)**			**Body appreciation (** ***Y*** **)**		
**Prediction variables**		***b***	**SE**		***b***	**SE**
Physical activity self-worth (X)	a	0.333^***^	0.079	c'	0.585^***^	0.088
Psychological resilience (M)	–	–	–	b	0.309^***^	0.037
Constant	IM	2.252	0.247	IY	1.285	0.281
	*R^2^* = 0.026			*R^2^* = 0.214		
	F_(1, 638)_= 17.694, *p* < 0.001			F_(2, 637)_= 74.237, *p* < 0.001		

^***^p < 0.001; SE: HC3 robust standard error, unstandardized coefficients (b) are reported.

## 4 Discussion

According to the main findings of the study, physical activity self-worth positively and significantly predicts psychological resilience, and psychological resilience is a significant predictor of body appreciation. Furthermore, it was determined that physical activity self-worth has a direct, positive, and statistically significant effect on body appreciation. In addition, the indirect effect of psychological resilience on the influence of physical activity self-worth on body appreciation was also found to be statistically significant. These findings indicate that physical activity self-worth affects body appreciation both directly and indirectly through psychological resilience. From a model perspective, it can be said that physical activity, self-worth, and psychological resilience together explain a significant amount of variance in body appreciation.

The finding that physical activity self-worth is significantly associated with psychological resilience is broadly consistent with prior research. Crocker and Knight (2005) emphasize that self-worth is closely linked to psychological wellbeing; this perspective provides a theoretical rationale for expecting domain-specific self-worth in physical activity to relate to individuals' psychological resources. In a similar vein, Özkara et al. (2016) reported a significant association between physical activity and psychological resilience. Qiu et al. (2025), in a study of university students, further showed that physical activity is related to psychosocial outcomes and that psychological resilience may operate as a mediating process within these associations. Taken together, this body of evidence suggests that higher physical activity self-worth is likely to co-occur with higher levels of psychological resilience, and that psychological resilience can be conceptualized as a plausible process variable when explaining how self-worth in the physical activity domain may translate into more favorable body-related evaluations. In addition, Zhibo et al. (2025) noted that physical activity–related self-worth may nurture psychological resources linked to resilience and may strengthen the extent to which these resources are reflected in indicators of wellbeing. Accordingly, the present result indicates that the association between physical activity self-worth and psychological resilience is a meaningful link to consider in models aiming to explain positive body-related outcomes among women.

A key finding of this study was that psychological resilience was significantly associated with body appreciation. This result is consistent with the broader resilience literature, which conceptualizes psychological resilience as the capacity to maintain psychological functioning and adapt effectively in the face of stressors and adverse experiences (Garmezy, 1991; Terzi, 2008). Individuals with higher resilience are described as more likely to recover following negative experiences, preserve a sense of control, and interpret change as less threatening (Kobasa, 1979; Kobasa et al., 1982). Such adaptive orientations may contribute to more stable self-evaluations and reduce the likelihood that stress-related experiences are translated into negative body-related appraisals. Accordingly, the present finding suggests that psychological resilience may be relevant not only for stress-related adjustment but also for understanding individual differences in positive body-related self-perceptions, including body appreciation.

Findings indicating a significant association between physical activity self-worth and body appreciation are broadly consistent with the existing literature. Singh and Sarraf (2025) noted that participation in physical activity is related to more favorable body-related evaluations and may coincide with higher physical self-worth, suggesting that exercise experiences can support perceptions of physical appearance, resilience, and competence. Raudsepp et al. (2013) reported a bidirectional relationship between physical activity and physical self-worth, implying that increases in activity may be accompanied by higher self-worth and that higher self-worth may, in turn, be linked to continued engagement. Consistent with this perspective, women who regularly participate in physical activity have been reported to be more satisfied with their bodies, to experience fewer appearance-related concerns, and to report higher self-worth than those who are not physically active (Guszkowska, 2015). Considering the activity context of the present sample, evidence that structured exercise practices are associated with meaningful differences in women's bodily and related outcomes further supports the plausibility of these links (Akçay et al., 2023). Taken together, the literature suggests that the value individuals attribute to themselves in the physical activity domain is closely related to positive body-related self-perceptions and may be relevant for understanding sustained participation patterns.

The study showed a significant indirect effect of physical activity self-worth on body appreciation through psychological resilience. Accordingly, higher physical activity self-worth was associated with higher psychological resilience, which, in turn, was associated with greater body appreciation. From a theoretical standpoint, Self-Determination Theory posits that intrinsic motivation and perceived competence in the physical activity domain are linked to psychological adjustment and resilience and may foster a more accepting, positive relationship with one's body (Deci and Ryan, 2000; Ryan and Deci, 2017). Consistent with this framework, prior research indicates that competence- and autonomy-supportive motivational processes in physical activity contexts are related to positive body-related outcomes and psychological wellbeing (Hagger and Chatzisarantis, 2007; Sabiston et al., 2019). Psychological resilience may be relevant in this pathway because it is associated with more adaptive coping and more stable self-evaluations under stress, which may reduce the likelihood that adverse experiences translate into negative body-related appraisals. Taken together, the present findings suggest that psychological resilience represents a meaningful process variable in understanding how physical activity self-worth is reflected in women's body appreciation.

## 5 Conclusion

In this study, a significant positive association was observed between physical activity self-worth and body appreciation among women. The mediation analysis indicated that physical activity self-worth significantly predicted psychological resilience, and psychological resilience, in turn, significantly accounted for variance in body appreciation. The finding that the direct effect of physical activity self-worth on body appreciation remained statistically significant after psychological resilience was included suggests that the relationship between these variables is more fully characterized when resilience is considered alongside self-worth. Taken together, the results position physical activity self-worth and psychological resilience as key psychosocial correlates of more favorable body-related evaluations in physically active women.

### 5.1 Limitations and strengths of the study

The findings of this study should be interpreted in light of several limitations. First, the sample was recruited through convenience sampling and was restricted to women attending pilates studios, zumba halls, and fitness centers in the Marmara Region of Türkiye. This sampling frame may limit the extent to which the results can be generalized to individuals living in other regions, those participating in different types of physical activity, or men. Second, because the study employed a cross-sectional design, causal conclusions cannot be drawn. Although the mediation model was statistically tested, the temporal ordering of the variables could not be established, and alternative explanations remain plausible. Third, the exclusive use of self-report measures increases vulnerability to social desirability bias, recall bias, and common method variance. Fourth, the combined use of face-to-face and online survey administration may have introduced mode-related differences in response behavior; however, such potential mode effects were not examined directly. Fifth, physical activity participation was operationalized in terms of weekly frequency, and the study did not include more granular indicators such as training intensity (e.g., perceived exertion or MET-based estimates), session duration and weekly volume, activity modality (e.g., resistance vs. aerobic training), structured regimen characteristics (e.g., periodization, load progression), or history of participation, nor did it incorporate device-based measures. Consequently, the role of these activity parameters in shaping the observed associations could not be evaluated in detail, and heterogeneity in training exposure may represent a source of residual confounding. Sixth, although the sample covered a relatively wide age range (18–69 years), age-stratified analyses were not conducted and age was not tested as a moderator. Therefore, the extent to which the observed associations generalize equally across different age groups remains unclear, and the magnitude and/or direction of the relationships may vary by age-related factors. Finally, because potentially relevant confounders such as body mass index, current psychological symptomatology, and health status were not comprehensively controlled, the possibility that the observed relationships were partly influenced by these factors should be acknowledged.

Despite these limitations, the study has notable strengths. The relatively large sample size (*N* = 640) supports statistical power and contributes to more stable estimation in the mediation model. Recruiting participants from four provinces and multiple exercise settings reduces reliance on a single site and strengthens the interpretability of the findings within the target population. From a methodological standpoint, testing the mediation hypothesis using Hayes' PROCESS macro (Model 4) with 5,000 bootstrap resamples provides a rigorous and widely accepted approach for evaluating indirect effects. Substantively, by jointly examining physical activity self-worth, psychological resilience, and body appreciation, the proposed model advances understanding of psychosocial processes that may underpin positive body-related outcomes among women. In this respect, the study offers a basis for theory-informed discussion and suggests practical relevance by indicating that psychological resilience may be a meaningful target in initiatives aimed at strengthening women's body-related experiences.

### 5.2 Recommendations for future studies and practitioners

The present findings indicate that physical activity self-worth is positively associated with body appreciation and that psychological resilience represents a meaningful mechanism through which this association operates. Future research should therefore address the main methodological constraints of the current design and further refine the proposed pathway. Longitudinal studies are recommended to establish temporal ordering and to examine whether changes in physical activity self-worth and psychological resilience predict subsequent changes in body appreciation over time. Intervention and experimental designs would provide stronger evidence regarding causality by testing whether programs intended to enhance physical activity self-worth and resilience produce measurable improvements in body appreciation. Because the sample was recruited via convenience sampling and was restricted to women attending pilates studios, zumba halls, and fitness centers in a specific region, future studies should recruit more diverse samples from different regions and socio-cultural contexts and extend recruitment to additional physical activity settings, such as outdoor recreation, community-based programs, and organized sport, to strengthen generalizability and allow contextual comparisons. Future research should also operationalize physical activity participation more comprehensively by assessing intensity, duration, type of activity, and length of prior engagement, and by incorporating objective indicators of activity where feasible. Given the reliance on self-report measures, subsequent work should consider strategies to reduce common method bias through multi-source data or alternative measurement approaches, and because data were collected via both face-to-face and online administration, potential mode effects should be tested explicitly, including measurement invariance across administration modes when appropriate. Finally, extending the model by including theoretically relevant constructs such as self-compassion, body functionality orientation, emotion regulation, perceived appearance pressures, social support, and instructor motivational climate may clarify the conditions under which psychological resilience most strongly contributes to body-related outcomes and may reveal additional pathways of influence.

From a practitioner perspective, the findings point to two practical targets in exercise settings: strengthening physical activity self-worth and supporting psychological resilience. Programs delivered in pilates studios, zumba halls, and fitness centers should be structured to cultivate mastery and competence experiences rather than emphasizing appearance-based outcomes. Instructors can facilitate this process by using graded progressions, individualized goal setting, and consistent feedback that highlights functional gains, skill development, and self-referenced improvement. Creating an environment in which participants experience competence and personal progress is likely to align with the observed link between physical activity self-worth and body appreciation. Because psychological resilience was associated with higher body appreciation, it may be beneficial to integrate brief resilience-supportive components into regular programming, particularly strategies that address common challenges such as setbacks, plateaus, performance concerns, and negative body-related thoughts. Practical, evidence-informed approaches include flexible and process-focused goal setting, coping planning for setbacks, adaptive self-talk, and cognitive reappraisal delivered in short structured formats before or after sessions. Additionally, instructors and program planners should foster a psychologically safe and inclusive climate that minimizes appearance-based comparisons and avoids communication that implicitly reinforces thin-ideal or appearance-centered goals, while emphasizing body functionality, health, and wellbeing. Monitoring program effectiveness using psychosocial indicators aligned with the tested mechanism, alongside physical outcomes, may further support evidence-informed practice by identifying participants who could benefit from additional mastery-based support and resilience-focused strategies and by guiding targeted refinements to program delivery.

## Data Availability

The raw data supporting the conclusions of this article will be made available by the authors, without undue reservation.
